# A new species of *Leodice* from Korean waters (Annelida, Polychaeta, Eunicidae)

**DOI:** 10.3897/zookeys.715.20448

**Published:** 2017-11-14

**Authors:** Hyun Ki Choi, Jong Guk Kim, Dong Won Kang, Seong Myeong Yoon

**Affiliations:** 1 National Marine Biodiversity Institute of Korea, Seocheon, Chungcheongnam-do 33662, Korea; 2 Marine Ecosystem and Biological Research Center, Korea Institute of Ocean Science and Technology, Busan 49111, Korea; 3 Department of Biology, College of Natural Sciences, Chosun University, Gwangju 61452, Korea

**Keywords:** COI, eunicid, Korea, polychaete, taxonomy

## Abstract

A new eunicid species, *Leodice duplexa* **sp. n.**, from intertidal and subtidal habitats in the eastern coast of South Korea is described. The new species is assigned to the C-2 group, and is similar to *Leodice antennata*, the type species of the genus, in having the following combination of characteristics: moniliform antennae and palps, bidentate compound falcigers, articulated peristomial and notopodial cirri, pectinate branchiae showing bimodal distribution of branchial filaments, and yellow aciculae. However, *L. duplexa* **sp. n.** is readily distinguished from *L. antennata* by the following features: the aciculae are 2–4 in number, with blunt or pointed tips and hammer-headed or bifid tips, and the subacicular hooks are paired in some chaetigers. A detailed description and illustrations are provided for the new species. The validity of the new species is also supported by a genetic comparison using sequences of the mitochondrial cytochrome c oxidase subunit I (COI). A revised key to known *Leodice* species is provided with a comparison of their morphological characteristics.

## Introduction

The genus *Leodice* Savigny in Lamarck, 1818, a member of genera belonging to family Eunicidae Berthold, 1827, had been previously considered an invalid taxon (Hsueh and Li 2014, Zanol et al. 2014). However, Zanol et al. (2014) proposed the validity of this genus based upon molecular and morphological data, and reinstituted the genus with 13 species previously reported as *Eunice* species: *Leodice americana* (Hartman, 1944), *L. antarctica* (Baird, 1869), *L. antennata* Savigny in Lamarck, 1818, *L. antillensis* (Ehlers, 1887), *L. gracilicirrata* Treadwell, 1922, *L. harassii* (Audouin & Milne Edwards, 1933), *L. limosa* (Ehlers, 1868), *L. lucei* (Grube, 1856), *L. marcusi* (Zanol et al., 2000), *L. miurai* (Carrera-Parra & Salazar-Vallejo, 1998), *L. rubra* (Grube, 1856), *L. thomasiana* (Augener, 1922), *L. torquata* (Quatrefages, 1866), and *L. valens* Chamberlin, 1919. Subsequently, *Eunice laurillardi* Quatrefages, 1866 was additionally treated as a species belonging to *Leodice* by Arias et al. (2015). The fauna of this group generally show a cosmopolitan distribution (Zanol et al. 2014); two *Leodice* species, *L. antennata* and *L. gracilicirrata*, have been reported as *Eunice* species in East Asia (Imajima and Hartman 1964, Imajima 1967, Miura 1977, 1986, Wu et al. 2013b; Hsueh and Li 2014).

Zanol et al. (2014) suggested that this genus has at least one of the following diagnostics: regularly articulated antennae and palps, light aciculae, light and bi- or tridentate subacicular hooks, and lateral black dots between posterior parapodia. Almost all species assigned to the A and C groups of Fauchald (1970) in *Eunice* species are expected to be *Leodice* species (Zanol et al. 2014). Here, the morphological diagnosis of *Leodice* described by Zanol et al. (2014) is followed.

While studying the polychaetes from Korean waters as a part of the ‘Securement, Analysis and Evaluation of Marine Invertebrate Bioresources’, a new species of the genus *Leodice* was found. In the present paper, detailed description and illustrations of the new species are provided with a genetic comparison between the new species and other *Leodice* species in the barcode region of the mitochondrial cytochrome c oxidase subunit I (COI). A taxonomic comparison of known *Leodice* species is also presented, with a revised taxonomic key based on the previous literature (Miura 1977, 1986, Fauchald 1992, Carrera-Parra and Salazar-Vallejo 1998, Zanol et al. 2000, Steiner et al. 2002, Zanol et al. 2007, Wu et al. 2013b, Zanol et al. 2014, Arias et al. 2015).

## Materials and methods

### Sampling and morphological observation

Samples were collected from rocky bottoms of the intertidal and subtidal zone. Specimens were sorted using sieves with pore size of 0.5 mm, fixed initially with 5% formaldehyde-seawater solution, and transferred to 85% ethyl alcohol. The characteristics of the whole body were observed with appendages dissected in a petri dish using dissection forceps or surgical knives and needles under stereomicroscope (SMZ1500; Olympus, Tokyo, Japan). Dissected specimens were mounted onto temporary slides using glycerol or permanent slides using polyvinyl lactophenol solution. Drawings were made under the stereomicroscope and light microscope (LABOPHOT-2; Nikon, Tokyo, Japan) with the aid of drawing tubes. Photographs were taken of the appendages in a permanent slide. Images of appendages were captured using an image system (i-SOLUTION/LITE, iMTechnology^®^, Vancouver, Canada). Specimens for scanning electron microscopy (SEM) were dehydrated by t-BuOH freeze dryer (VFD-21S; Vacuum Device, Ibaraki, Japan). They were mounted on stubs and coated with gold-palladium. SEM observations were carried out using a scanning electron microscope (SU3500; Hitachi, Tokyo, Japan). Type material and additional material examined were deposited in the National Marine Biodiversity Institute of Korea (MABIK).

### Molecular analysis

Genomic DNA was extracted from the posterior segments of three specimens selected among additional materials using a DNeasy Blood and Tissue Kit (Qiagen, Hilden, Germany) according to the manufacture’s protocol. Amplifications of partial sequences of the mitochondrial cytochrome c oxidase subunit I (COI) from gDNA were carried out by polymerase chain reaction (PCR) method using a set of primers: ACOIAF 5’- CWAATCAYA AAGATATTGGAAC-3’ and ACOIAR 5’- AATATAWACTTCWGGGTGACC -3’ (Zonal et al. 2010). PCR amplification was conducted in a total volume of 20 µL: 10 µL of 2x DyeMIX-Tenuto (Enzynomics), 0.5 µL of each primer, 1 µL of gDNA, and 8 µL of sterile water. PCR condition was determined based on the work of Zonal et al. (2010) as follows: 5 min at 94°C, followed by 35 cycles of 1 min at 94°C, 1 min at 45°C, and 2 min at 72°C, with a final extension of 7 min at 72°C. PCR products were purified with a QIAquick® PCR Purification Kit (Qiagen, Chatsworth, CA, USA). Sequences for the new species were obtained by an Applied Biosystems 3730 DNA sequencer, and deposited in GenBank under accession number MF669544–MF669546. These sequences were aligned with those of other *Leodice* species and outgroup taxa using Geneios Pro v.9.1.8 (Biomatters, Auckland, New Zealand). The genetic distances of the new species from other species and the phylogenetic tree of them were produced by MEGA v.6.06 (Tamura et al., 2013).

## Systematic accounts

### Family Eunicidae Berthold, 1827

#### Genus *Leodice* Savigny in Lamarck, 1818

##### Leodice duplexa sp. n.

Taxon classificationAnimaliaEunicidaEunicidae

http://zoobank.org/64134EF0-FFE9-4FB3-8C64-4030C310ED09

Figs 1 , 2

###### Type locality.

South Korea, Gyeongsangbuk-do Province: Gyeongju-si County, Gampo-eup, Oryu 1-ri, 35°48'13"N, 129°32'21"E, intertidal rocky bottom.

###### Material examined.

***Holotype*.** complete specimen (53.0 mm long, 4.8 mm width), cat no. MABIKNA00146045. ***Paratypes*.** one complete specimen (24.0 mm long, 4.9 mm width), cat no. MABIKNA00146046; one incomplete specimen (15.0 mm long, 3.3 mm width), cat no. MABIKNA00146047. All type material was collected from intertidal rocky bottom at the type locality on 9 April 2014.

###### Additional material.

South Korea, Gyeongsangbuk-do Province: 5 specimens, Ulleung-gun County, Ulleung-eup, Dokdo-ri, 37°14’31”N, 131°52’06”E, 05 Sep. 2016, subtidal rocky bottom at 10–15 m depth; 5 specimens, Pohang-si County, Homigot-myeon, Guman-ri, 36°04'35"N, 129°34'31"E, 19 May 2015; 2 specimens, Yeongdeok-gun County, Chuksan-myeon, Gyeongjeong-ri, 36°27'40"N, 129°32'34"E, 17 Sep. 2014., intertidal rocky bottom.

###### Diagnosis.

Prostomium with three antennae and one pair of lateral palps arranged in crescent pattern; palpostyles and ceratostyles regularly articulated and moniliform, and with ring-shaped palpo- and ceratophores. Peristomial cirri with four weak articulations, not extending middle part of first peristomial ring. Pectinate branchiae from chaetiger VI to near posterior end, with maximum of 7–8 branchial filaments. Limbate chaetae slender, with narrow wings. Heterodont pectinate chaetae with 5–10 teeth. Compound falcigers bidentate, with hoods marginally serrated. Aciculae yellow, with both blunt and hammer-headed or bifid tip, and 2–4 per parapodium. Subacicular hooks yellow, tridentate, present from chaetiger XXIII to last chaetiger, and 1–2 per parapodium. Pygidium with two pairs of anal cirri with four articulations.

###### Description.

Holotype: complete specimen with cylindrical body, slightly flattened dorsoventrally in posterior segments, and with approximately 94 segments.

**Figure 1.**
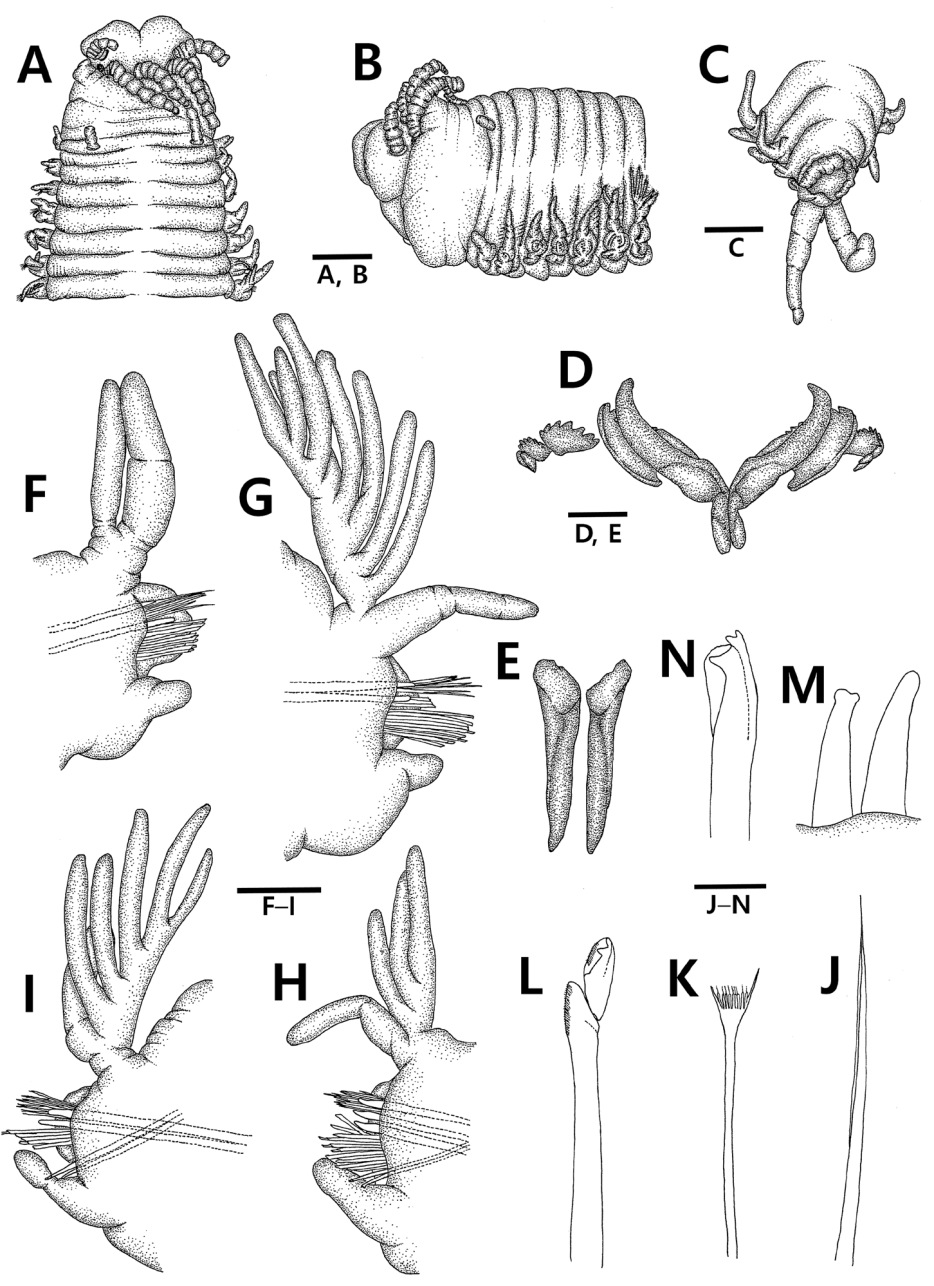
*Leodice duplexa* sp. n., **A–C, F–N** holotype (MABIKNA00146045) **D, F** paratype (MABIKNA00146046) **A** anterior end, dorsal view **B** anterior end, lateral view **C** posterior end, dorsal view **D** maxillae **E** mandible **F** left parapodium VI, anterior view **G** left parapodium XX, anterior view **H** right parapodium XXXVIII, anterior view **I** right parapodium LX, anterior view **J** limbate chaeta on parapodium XLVII **K** pectinate chaeta on parapodium XLVII **L** compound falciger on parapodium XLVII **M** subacicular hook on parapodium XXXVIII **N** aciculae with blunt and hammer-headed tips on parapodium LXXXV. Scale bars: 1.0 mm (**A, B**), 0.5 mm (**C–E**), 0.4 mm (**F–I**), 0.05 mm (**J–N**).

Prostomium bilobed, distinctly shorter and narrower than peristomium, and about as deep as 1/2 of peristomium; prostomial lobes anteriorly rounded, dorsally flattened, separated by shallow and narrow notch. Prostomial appendages consisted of three antennae and two lateral palps, arranged in shallow semicircle, evenly spaced, similar in thickness; palpophores and ceratopores ring-shaped without articulation; palpostyles and ceratostyles tapering, regularly articulated, with up to 12 moniliform articulations in A-I extending to anterior edge of chaetiger I, but incomplete distally with 8–11 moniliform articulations in others except A-I (Fig. 1A, B).

Eyes black, spherical, and located between bases of palps and lateral antennae (Fig. 1A).

Peristomium cylindrical, divided into first and second ring; first ring 3–4 times longer than second one; peristomial cirri with four weak articulations, not extending middle part of first peristomial ring (Fig. 1A, B).

Maxillary formula (in paratype): Mx I 1+1; Mx II 6+6; Mx III 7+0; Mx IV 6+7; Mx V 1+1; Mx III located at frontal end of distal arc with left Mx IV. Mandibles flat (Fig. 1D, E).

Branchiae pectinate, slightly longer than dorsal cirri, and present on more than 65% of total number of chaetigers from chaetiger VI to near posterior end. Branchial filaments bimodal distribution, single at first branchial chaetiger, reaching maximum of eight in number between chaetigers IX to XXIII, reduced to 3–4 in number on chaetigers XXXI to XLVIII, increasing to 5–6 in number on chaetigers XLIX to LXIV, and thereafter decreasing to 2–4 in number on posterior chaetigers. Last six chaetigers without branchiae (Fig. 1F–I).

Dorsal cirri tapering and digitiform, with 2–3 weak articulations (Fig. 1F–I).

Anterior neuropodial lobes truncate and distally rounded with aciculae emerging near midline; pre- and postchaetal lobes low, transverse folds. Ventral cirri on anterior chaetigers thick and ovoid-shaped, with slightly inflated base; median ones with inflated base more than anterior ones; posterior ones slightly elongated with smaller base than median ones (Fig. 1F–I).

**Figure 2.**
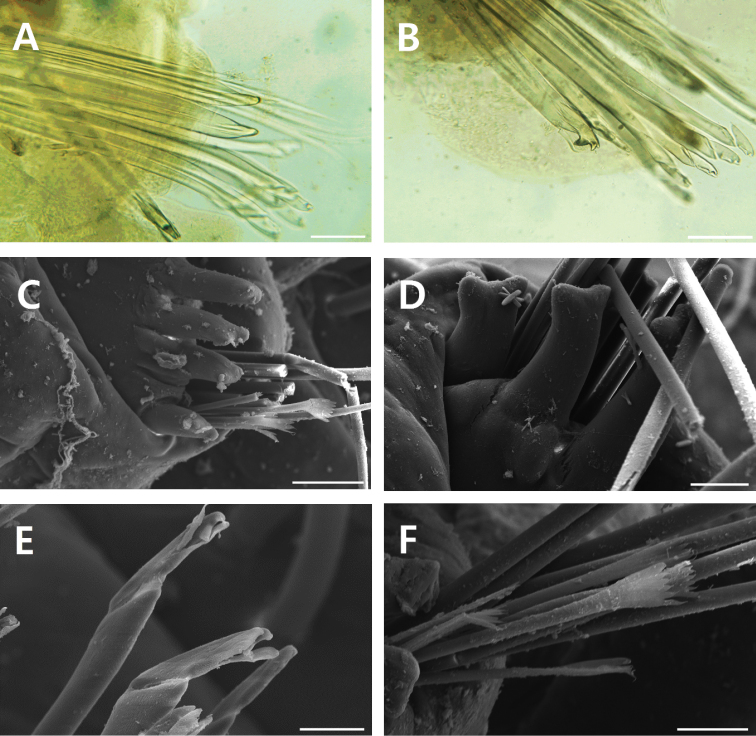
*Leodice duplexa* sp. n., **A–D** paratype (MABIKNA00146047) **A** yellow aciculae with pointed tips **B** yellow and paired subacicular hooks **C** parapodium with four aciculae **D** aciculae with hammer-headed or bifid tips **E** compound falcigers with bidentate appendages **F** pectinate chaeta. Scale bars: 0.05 mm (**A–C**), 0.025 mm (**D–F**).

Limbate chaetae slender and elongate, longer than other chaetae, and with narrow wings (Fig. 1J). Pectinate chaetae flaring and with marginal teeth and 4–9 inner teeth (Figs 1K, 2F). Compound falcigers with distally inflated shafts and bidentate appendage; shafts marginally serrated on inflated region; appendages tapering, slender, with both proximal tooth directed laterally and distal tooth gently curved and directed upwardly, and proximal tooth slightly smaller than distal tooth on anterior chaetigers, but larger than distal tooth on posterior chaetigers; guards marginally serrated, without mucros (Figs 1L, 2E). Pseudocompound falcigers and compound spinigers absent. Aciculae yellow, straight, tapering with both blunt or pointed tips and hammer-headed or bifid tips, rounded in cross-section, and 2–3 in number per parapodium (maximum of four in paratype and more than two aciculae usually appeared in posterior parapodia); separation between core and sheath indistinct in aciculae and subacicular hooks (Figs 1M, 2A, C, D). Subacicular hooks yellow, tridentate, present from chaetiger XXIII to last chaetiger, and 1–2 in number per parapodium; shaft straight, subdistally tapering; proximal tooth triangle, distally blunt, directed laterally, larger than distal teeth; guards covering only proximal tooth (Figs 1N, 2B).

Pygidium with two pairs of anal cirri; dorsal pair as long as last five chaetigers with up to four cylindrical articulations, ventral pair reduced to small bump (Fig. 1C).

###### Etymology.

The epithet of the specific name, *duplexa*, is derived from the Latin *duplex*, meaning ‘double’. This name refers to the presence of paired subacicular hooks.

###### Distribution.

The East Sea (or the Sea of Japan) of South Korea.

###### Remarks.

*Leodice* species were redefined by Zanol et al. (2014), but their specific characteristics were insufficiently dealt in that work. However, diagnostic features of many *Leodice* species have been well studied under the names of *Eunice* species by the previous works (Hartman 1944, Day 1967, Miura 1977, 1986, Fauchald 1992, Carrera-Parra and Salazar-Vallejo 1998, Zanol et al. 2000, Steiner et al. 2002, Zanol et al. 2007, Wu et al. 2013b). The new species of the present study can be discriminated from its relatives by the distinguishing morphological features provided by these previous works. *Leodice duplexa* sp. n. could be classified into the C-2 group of Fauchald (1970, 1992), because it has translucent and tridentate subacicular hooks and the branchiae are arising from the chaetigers more than 65% of a total number of them. Among the species assigned to this group, *L. duplexa* sp. n. is closely related to *L. antennata*, the type specie of *Leodice*, in the following morphological features: the antennae and palps are regularly articulated and moniliform; the compound falcigers are bidentate; the peristomial and notopodial cirri are articulated; the branchiae are pectinate and show the bimodal distribution of branchial filaments; the aciculae are yellow (Hartman 1944, Day 1967, Imajima 1967, Miura 1977, 1986, Fauchald 1970, 1992, Zanol et al. 2007, Wu et al. 2013b). Nonetheless, the new species is distinguishable from *L. antennata* in two points as follows: the new species has 2–4 aciculae, while *L. antennata* bears one pair; the subacicular hooks are paired in some chaetigers in the new species, but always single in each chaetiger in *L. antennata* (Day 1967, Imajima 1967, Miura 1977, Fauchald 1992, Zanol et al. 2007, Wu et al. 2013b).

The distal shape of aciculae has been considered as a useful character for distinguishing eunicid species (Miura 1986, Fauchald 1992, León-González et al. 2004, Zanol et al. 2007, Wu et al. 2013a, Hsueh and Li 2014). The new species shows aciculae with blunt or pointed tips and hammer-headed or bifid tips, which are clearly differentiated from the aciculae with blunt or pointed tips of *L. antennata* described from the Gulf of Suez, its type locality (Fauchald 1992, Zanol et al. 2007). However, the appearance of the aciculae with hammer-headed or bifid tips, which is found in the new species, has been frequently mentioned in the previous descriptions of *L. antennata* (Hartman 1944, Day 1967, Imajima, 1967, Miura 1977, 1986). Despite the discrepancy among the previous descriptions of *L. antennata*, we accepted that the difference in terms of the distal shape of aciculae between *L. duplexa* sp. n. and *L. antennata* is valid, based on the description of *L. antennata* from the type locality by Fauchald (1992) and Zanol et al. (2007). We think that the taxonomic validity of the previous descriptions of *L. antennata*, including the presence of aciculae with hammer-headed or bifid tips, is questionable (Hartman 1944, Day 1967, Imajima 1967, Miura 1977, 1986) and they could be assigned to another species or subspecies through further study and detailed comparison with the materials from the type locality.

The aciculae with both blunt or pointed and hammer-headed or bifid tips, which appear in the new species, have been often described in the eunicid species. In the species of the C-2 group of the eunicids (Fauchald 1970, 1992), this feature is known from three *Eunice* species (which might be *Leodice* species), *E. aedificatrix* Monro, 1933, *E. ornata* Andrews, 1891, *E. uschakovi* Wu, Sun & Liu, 2013, and from two *Leodice* species, *L. rubra* (Grube, 1856) and *L. lucei* (Grube, 1856) (Fauchald 1992, Wu et al. 2013a). Among them, *L. rubra* is especially similar to *L. duplexa* sp. n. in the presence of paired subacicular hooks in some chaetigers (Fauchald 1992, Steiner et al. 2002). However, these two species differ from each other by the number of branchial filaments and aciculae: *L. duplexa* sp. n. is with a maximum of eight branchial filaments and 2–4 aciculae per parapodium, whereas *L. rubra* displays a maximum of 21 filaments and single paired aciculae (Fauchald 1992, Steiner et al. 2002). Meanwhile, *L. duplexa* sp. n. resembles *L. valens* in the number of aciculae and subacicular hooks. However, these two species are distinguishable from each other because *L. valens*, which is regarded as the member of the A-1 group by Fauchald (1970, 1992), has translucent and bidentate subacicular hooks (Fauchald 1992), while *L. duplexa* sp. n. has translucent but tridentate subacicular hooks. Additionally, the branchiae are present on less than 55% of chaetigers in *L. valens* (Fauchald 1992), but on more than 65% in the new species.

###### Genetic comparison.

**Figure 3.**
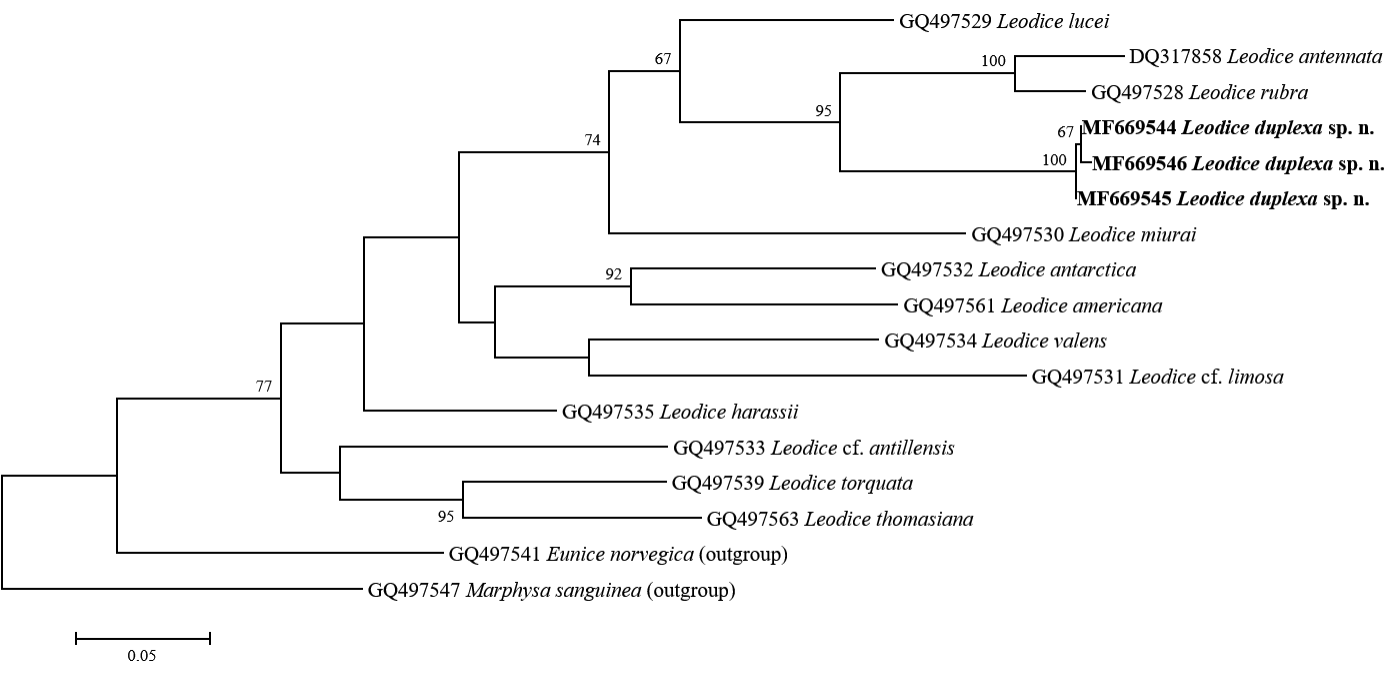
Maximum likelihood tree showing phylogenetic relationship based on COI sequences of 13 *Leodice* species with two outgroup taxa (*Eunice norvegica* and *Marphysa sanguinea*).

We obtained three partial COI sequences of a total 664 bp size from three individuals of *Leodice duplexa* sp. n., respectively. All COI sequences obtained are identical. For the genetic comparison on the new species, we sort out the sequences of 14 eunicid species including 12 *Leodice* species, which were originally registered as *Eunice* species, and two non-*Leodice* species as outgroup taxa, *Eunice norvegica* (Linnaeus, 1767) and *Marphysa sanguinea* (Montagu, 1813), from GenBank (Schulze 2006, Zanol et al. 2010). The genetic distances between the new species and these 14 eunicid species measured by Kimura-2-parameter model are represented in Table 2. *Leodice duplexa* sp. n. is distinguishable from previously described 12 *Leodice* species in that the inter-specific distances between the new species and other *Leodice* species are distinct with the ranges from 8.2 to 14.4%. Among *Leodice* species, *L. duplexa* sp. n. is turned out to be closely related to *L. lucei* and mostly distinguished from L. cf. antillensis (Table 2). In Maximum likelihood (ML) tree based on these genetic data (Fig. 3), the new species is contained within *Leodice* species. Especially, *L. duplexa* sp. n. belongs to a clade with *L. antennata*, *L. rubra*, *L. lucei*, and *L. miurai*, and they share several significant morphological characteristics such that the antennae and palps are regularly moniliform and the subacicular hooks are yellow and tridentate (Fauchald 1992, Carrera-Parra and Salazar-Vallejo 1998, Steiner et al. 2002, Zanol et al. 2007). Conclusively, the result of the genetic analysis could support the validity of the new species identified by the morphological differences from its congeners.

**Table 1.** Comparison of morphological characteristics among known *Leodice* species.

Species	Eyes	Articulations of antennae and palpal styles	Peristomial cirri	Branchiae				Notopodial cirri	Subacicular hooks			Compound falcigers	Aciculae		Data source
				Shape	Starting chaetiger	Distribution	Number of filaments		Shape	Starting chaetiger	Number per parapodium		shape	Number per parapodium	
*L. americana* (Hartman 1944)	present	regularly cylinderical	smooth	pectinate	3	less than 55% of body	2–20	articulated	yellow, tridentate	25	single	bidentate, with mucros	yellow, bently blunt tips	paired	Fauchald 1992
*L. antarctica* (Baird, 1869)	absent	irregularly cylinderical	smooth	pectinate	3	less than 55% of body	1–5	articulated	yellow, bidentate	31–44	single	bidentate	yellow, blunt tips	paired	Fauchald 1992
*L. antennata* Lamarck, 1818	present	moniliform	with articulations	pectinate	5–7	more than 65% of body	2–7	articulated	yellow, tridentate	24–26	single	bidentate	yellow, blunt or pointed tips	paired	Fauchald 1992, Zanol et al. 2007
*L. antillensis* (Ehlers, 1887)	present	irregularly cylinderical	with articulations	pectinate	4	less than 55% of body	1–6	articulated	yellow, bidentate	33	1–2	bidentate	yellow, flattened and expanded tips	paired	Fauchald 1992
*L. duplexa* sp. n.	present	moniliform	with articulations	pectinate	5–6	more than 65% of body	1–8	articulated	yellow, tridentate	24–26	1–2	bidentate	yellow, both blunt and hammer-headed or bifid tips	2–4	Present study
*L. gracilicirrata* Treadwell, 1922	unknown	irregularly cylinderical	with articulations	pectinate	3	less than 55% of body	1–7	articulated	yellow, bidentate	63	single	bidentate	yellow, both pointed with mucros and bluntly rounded tips	paired	Fauchald 1992, Miura 1986
*L. harassii* (Audouin & Milne Edwards, 1933)	faintly present	regularly cylinderical	smooth	pectinate	4	more than 65% of body	1–10	smooth	light brown, bidentate	28	single	bidentate	yellow, turned dark brown, pointed tips	paired	Fauchald, 1992, Zanol et al. 2007
*L. laurillardi* (Quatrefages, 1866)	present	regularly cylinderical	with articulations	pectinate	2–3	more than 65% of body	1–10	articulated	dark brown, bidentate	29–32	single	bidentate	dark brown, blunt tips	paired	Fauchald, 1992, Arias et al. 2015
*L. limosa* (Ehlers 1868)	unknown	regularly cylinderical	smooth	pectinate	3	less than 55% of body	1–12	smooth	yellow, tridentate	before 30	unknown	bidentate, with mucros	yellow, blunt tips	unknown	Fauchald 1992
*L. lucei* (Grube, 1856)	present	moniliform	with articulations	pectinate	5	more than 65% of body	1–8	articulated	yellow, tridentate	24–34	single	bidentate, with mucros	yellow, distinctly hammer-headed or bifid tips	paired	Fauchald 1992
*L. marcusi* (Zanol et al., 2000)	present	moniliform	with articulations	palmate	4	more than 65% of body	1–4	articulated	black, bidentate	19–26	single	bidentate	black, pointed tips	paired	Zanol et al. 2000
*L. miurai* (Carrera-Parra & Salazar-Vallejo, 1998)	present	moniliform	with articulations	pectinate	5–6	less than 55% of body	1–3	articulated	yellow, tridentate	18–23	single	bidentate to tridentate	black, bifid tips	unknown	Carrera-Parra & Salazar-Vallejo 1998, Zanol et al. 2007
*L. rubra* (Grube, 1856)	present	moniliform	with articulations	pectinate	4–6	more than 65% of body	1–21	articulated	yellow, tridentate	27	1–2	bidentate	yellow, both pointed or blunt and bifid tips	paired	Fauchald 1992, Steiner et al. 2002, Zanol et al. 2007
*L. thomasiana* (Augener, 1922)	present	basally cylindrical and distally ovate	with articulations	palmate	4	more than 65% of body	1–3	articulated	brown, bidentate	22–24	1–2	bidentate	brown, blunt tips	paired	Fauchald 1992, Zanol et al. 2007
*L. torquata* (Quatrefages, 1866)	present	moniliform	with articulations	pectinate	3	more than 65% of body	2–7	articulated	brown, bidentate	22	1–2	bidentate	brown, blunt tips	paired	Fauchald 1992, Zanol et al. 2007
*L. valens* Chamberlin, 1919	present	regularly cylinderical	with articulations	pectinate	3	less than 55% of body	1–11	articulated	yellow, bidentate	43	1–2	bidentate	yellow, blunt tips	2–4	Fauchald 1992

**Table 2.** Genetic distance (K2P) based on 664 bp size of COI sequence among 13 *Leodice* species with two outgroup taxa (*Eunice norvegica* and *Marphysa sanguinea*).

No.	Species	Accession No.	1	2	3	4	5	6	7	8	9	10	11	12	13	14	15	16	Data source
**1**	*Leodice duplexa* sp. n.	MF669544																	Present study
**2**	*Leodice duplexa* sp. n.	MF669545	0.000																"
**3**	*Leodice duplexa* sp. n.	MF669546	0.000	0.000															"
**4**	*L. americana*	GQ497561	0.095	0.095	0.095														Zanol et al. 2010
**5**	*L. antarctica*	GQ497532	0.088	0.088	0.088	0.043													"
**6**	*L. antennata*	DQ317858	0.097	0.097	0.097	0.103	0.124												Schulze 2006
**7**	L. cf. antillensis	GQ497533	0.144	0.144	0.144	0.102	0.088	0.159											Zanol et al. 2010
**8**	*L. harassii*	GQ497535	0.108	0.108	0.108	0.062	0.043	0.115	0.095										"
**9**	L. cf. limosa	GQ497531	0.124	0.124	0.124	0.104	0.110	0.102	0.153	0.116									"
**10**	*L. lucei*	GQ497529	0.082	0.082	0.082	0.088	0.095	0.062	0.115	0.088	0.102								"
**11**	*L. miurai*	GQ497530	0.104	0.104	0.104	0.109	0.116	0.096	0.145	0.123	0.153	0.083							"
**12**	*L. rubra*	GQ497528	0.083	0.083	0.083	0.089	0.109	0.012	0.144	0.101	0.102	0.062	0.111						"
**13**	*L. thomasiana*	GQ497563	0.143	0.143	0.143	0.094	0.081	0.136	0.108	0.068	0.129	0.128	0.150	0.121					"
**14**	*L. torquata*	GQ497539	0.114	0.114	0.114	0.094	0.068	0.121	0.094	0.055	0.144	0.128	0.135	0.107	0.024				"
**15**	*L. valens*	GQ497534	0.116	0.116	0.116	0.089	0.069	0.139	0.075	0.075	0.103	0.095	0.132	0.124	0.129	0.115			"
**16**	*Eunice norvegica* (outgroup)	GQ497541	0.188	0.188	0.188	0.143	0.121	0.165	0.150	0.129	0.157	0.180	0.180	0.165	0.144	0.129	0.143		"
**17**	*Marphysa sanguinea* (outgroup)	GQ497547	0.172	0.172	0.172	0.135	0.101	0.157	0.115	0.101	0.150	0.121	0.157	0.142	0.136	0.121	0.114	0.101	"

### Key to known species of the genus *Leodice* (based on Fauchald 1992 and Zanol et al. 2014)

**Table:** 

1	Antennae and palps regularly articulated	**2**
–	Antennae and palps irregularly articulated	**13**
2	Subacicular hooks bidentate	**3**
–	Subacicular hooks tridentate	**7**
3	Peristomial cirri and notopodial cirri articulated	**4**
–	Peristomial cirri and notopodial cirri smooth	***L. harassii* (Audouin & Milne Edwards, 1933)**
4	Branchiae present on more than 65% of body	**5**
–	Branchiae present on less than 55% of body	***L. valens* Chamberlin, 1919**
5	Parapodia with pectinate branchiae	**6**
–	Parapodia with palmate branchiae	***L. marcusi* (Zanol et al., 2000)**
6	Subacicular hooks usually paired	***L. torquata* (Quatrefages, 1866)**
–	Subacicular hooks always single	***L. laurillardi* (Quatrefages, 1866)**
7	Branchiae with up to 20–21 filaments	**8**
–	Branchiae with less than 12 filaments	**9**
8	Aciculae with bifid or hammer-headed tips absent	***L. americana* (Hartman, 1944)**
–	Aciculae with bifid or hammer-headed tips present	***L. rubra* (Grube, 1856)**
9	Guards of compound falcigers with mucros	**10**
–	Guards of compound falcigers without mucros	**11**
10	Palpostyles and ceratostyles cylindrical; peristomial cirri smooth	***L. limosa* (Ehlers 1868**
–	Palpostyles and ceratostyles moniliform; peristomial cirri articulated	***L. lucei* (Grube, 1856)**
11	Compound falcigers tridentate in posterior chaetigers	***L. miurai* (Carrera-Parra & Salazar-Vallejo, 1998)**
	Compound falcigers only bidentate	**12**
12	Subacicular hooks always single; aciculae paired	***L. antennata* Lamarck, 1818**
–	Subacicular hooks paired in some chaetigers; aciculae 2–4 in number	***L. duplexa* sp. n.**
13	Subacicular hooks and acicular light; branchiae pectinate	**14**
–	Subacicular hooks and acicular dark; branchiae palmate	***L. thomasiana* (Augener, 1922)**
14	Subacicular hooks paired in some chaetigers	***L. antillensis* (Ehlers, 1887)**
–	Subacicular hooks always single	**15**
15	With finely hooded aciculae	***L. gracilicirrata* Treadwell, 1922**
–	Without finely hooded aciculae	***L. antarctica* (Baird, 1869)**

## Supplementary Material

XML Treatment for Leodice duplexa
